# Supplementary material for: Spatial profiling of non-small cell lung cancer provides insights into tumorigenesis and immunotherapy response
Source: Commun Biol. 2024 Aug 2;7:930. doi: 10.1038/s42003-024-06568-w (PMC11297140; doi:10.1038/s42003-024-06568-w)

Supplementary Figure 1: Spatial characteristics of NSCLC. **(a)** A representative microscopic images of LUAD and LUSC tissues. **(b - d)** ESTIMATE analysis of all NSCLC, LUSC, LUAD tissues. *$p < 0.05$, **$p < 0.01$, *** $p < 0.005$, *** $p < 0.001$. NSCLC non-small cell lung cancer, LUSC lung squamous cell carcinoma, LUAD lung adenocarcinoma, ESTIMATE Estimation of STromal and Normal tissues in Malignant Tumor tissues using Expression data. Box plot components: The center line of the box represents the median, and the upper and lower limits of the box represent the upper and lower quantiles, respectively. Whiskers indicate a value of 1.5 times the interquartile range.

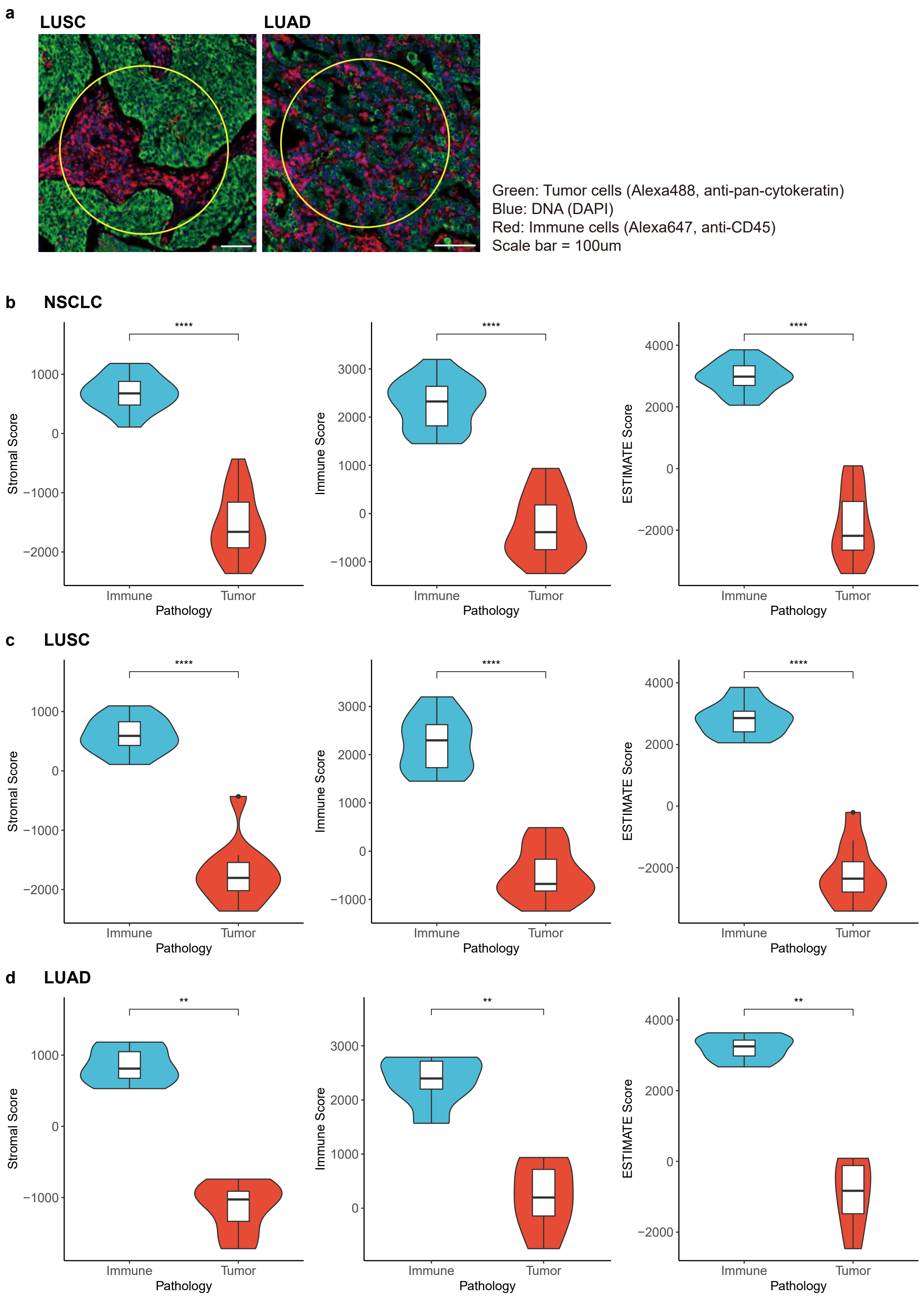

Supplementary Figure 2: Expression profiling and molecular characteristics of NSCLC transcriptome by pathologic grade. **(a - c)** Volcano plot of DEGs between **(a)** NSCLC (n = 18), **(b)** LUSC (n = 11) and **(c)** LUAD (n = 6) tumor and immune samples. Only genes with adjusted *p*-value <= 0.05 were considered DEGs. **(d)** Dotplot showing GSEA results using MSigDB hallmark gene sets between NSCLC tumor and immune samples. **(e)** Dotplot showing top 20 GSEA results using MSigDB KEGG gene sets between NSCLC tumor and immune samples. **(f)** Dotplot showing top 20 GSEA results using MSigDB GO BP gene sets between NSCLC tumor and immune samples. **(g)** Dotplot showing GSEA results using MSigDB celltype signature gene sets between NSCLC tumor and immune samples. DEGs differentially expressed genes, GSEA gene set enrichment analysis, MSigDB molecular signature database, GO BP gene ontology biologic process, NES normalized enrichment score.

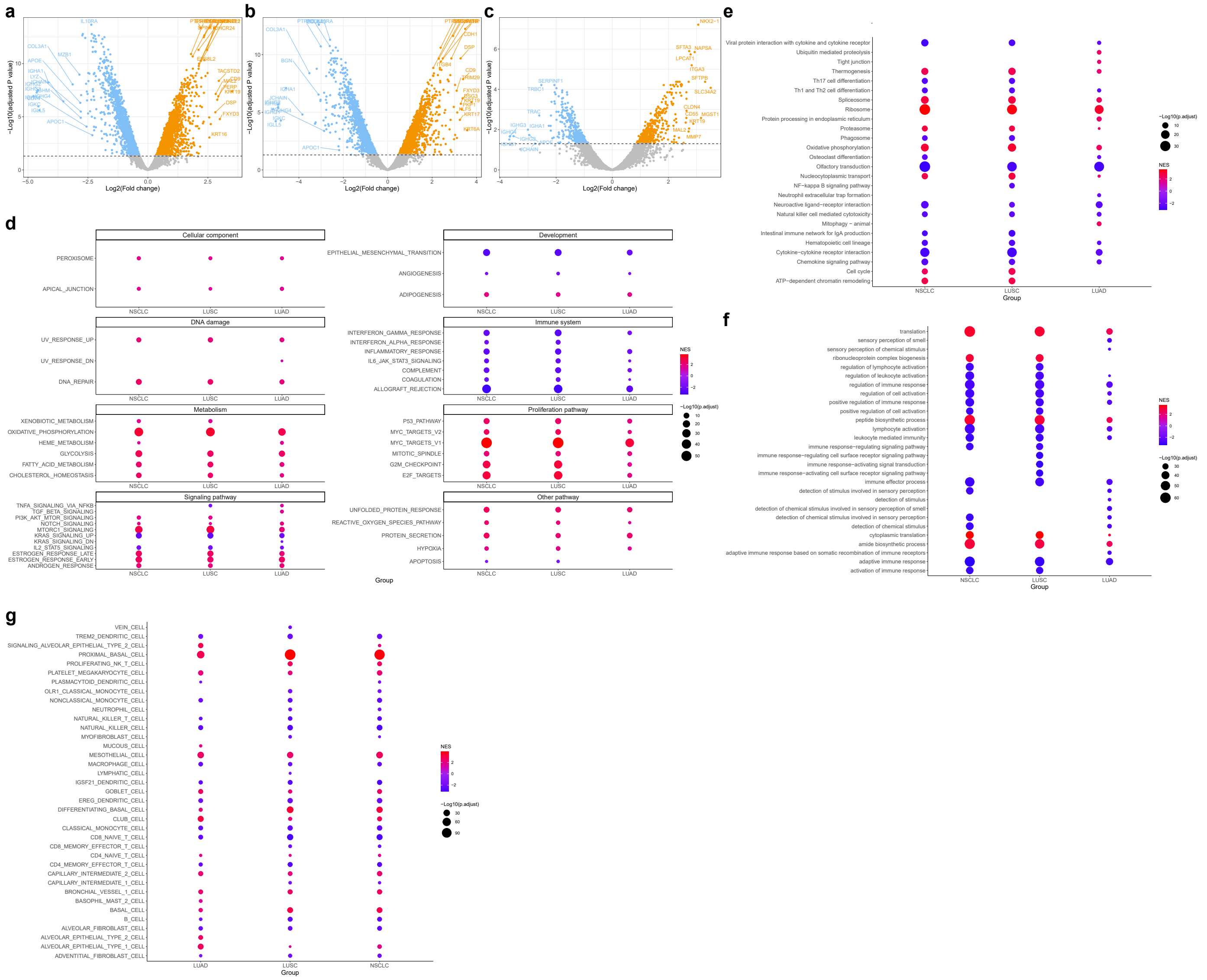

Supplementary Figure 3: The heatmap of module-trait relationship with response, diagnosis and pathologic grade by WGCNA. **(a)** The heatmap of module-trait relationship in all NSCLC samples (n = 36). **(b)** The heatmap of module-trait relationship in tumor samples. (n = 18) **(c)** The heatmap of module-trait relationship in immune samples (n = 18). The numbers in each cell of the heatmap were displayed in the form of a correlation coefficient (p-value). WGCNA weighted correlation network analysis.

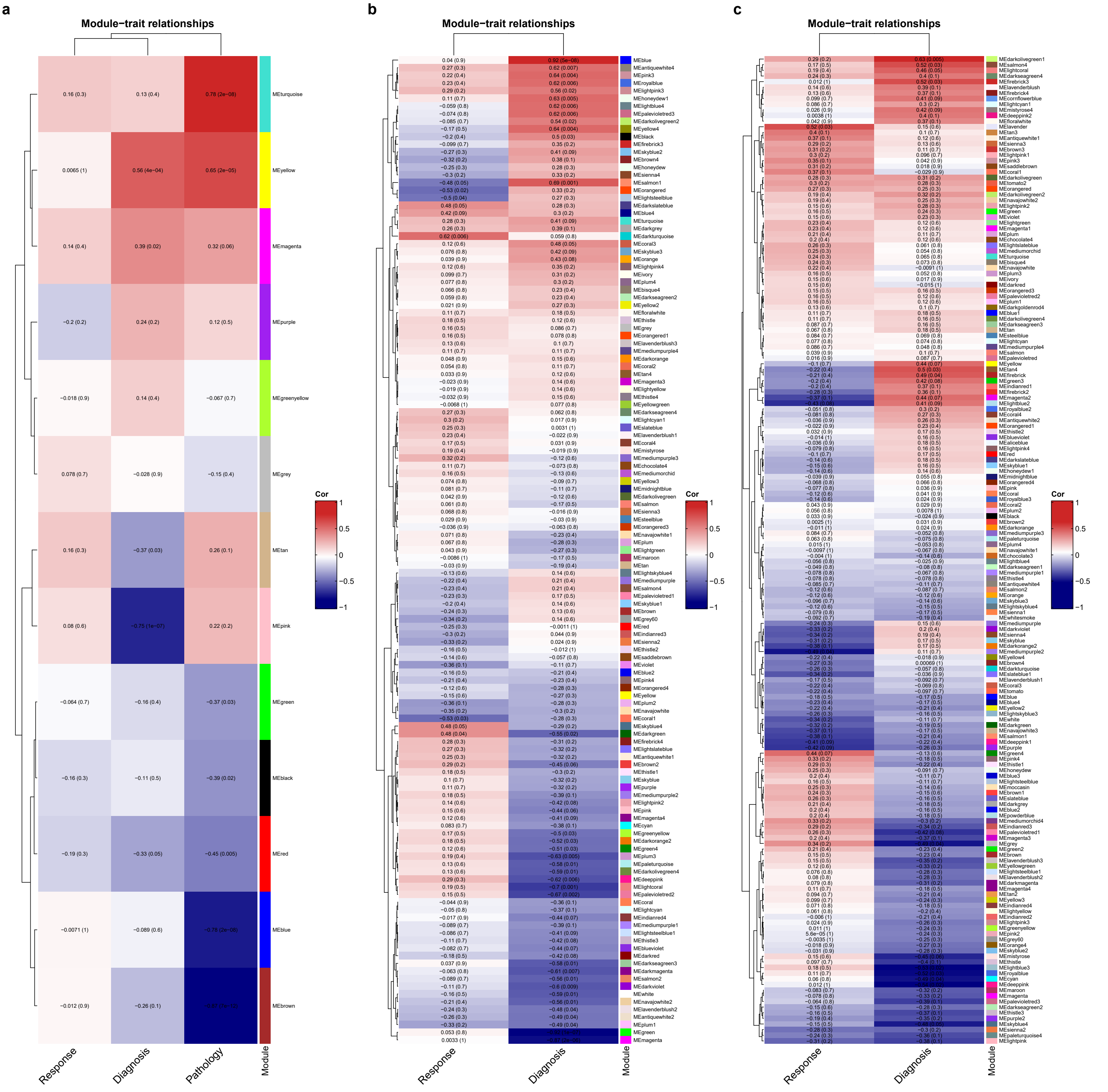

Supplement: Supplementary file 2 — Supplementary Information [file 42003_2024_6568_MOESM2_ESM.pdf]
