# Supplementary material for: Spatial profiling of non-small cell lung cancer provides insights into tumorigenesis and immunotherapy response
Source: Commun Biol. 2024 Aug 2;7:930. doi: 10.1038/s42003-024-06568-w (PMC11297140; doi:10.1038/s42003-024-06568-w)
Supplement: Supplementary file 3 — Description of Additional Supplementary Files [file 42003_2024_6568_MOESM3_ESM.docx]

**Description of Additional Supplementary Files**

**File name:** Supplementary Data 1

**Description:** ESTIMATE analysis results of NSCLC tissues.

**File name:** Supplementary Data 2

**Description:** Transcriptome expression profiling between NSCLC tumor and immune samples

**File name:** Supplementary Data 3

**Description:** Transcriptome expression profiling of NSCLC immune checkpoint blockade responsiveness

**File name:** Supplementary Data 4

**Description:** Transcriptome expression profiling of LUSC immune checkpoint blockade responsiveness

**File name:** Supplementary Data 5

**Description:** Transcriptome expression profiling of LUAD immune checkpoint blockade responsiveness

**File name:** Supplementary Data 6

**Description:** NMF signatures activity, validation and ORA results

**File name:** Supplementary Data 7

**Description:** WGCNA validation and ORA results

**File name:** Supplementary Data 8

**Description:** B cell subtype markers, expression of B cell subtype markers, and validation
